# Allergic reactions to genus *Morus* plants: a review

**DOI:** 10.1186/s12948-020-00116-7

**Published:** 2020-02-19

**Authors:** F. Papia, C. Incorvaia, L. Genovese, S. Gangemi, P. L. Minciullo

**Affiliations:** 1 Allergy and Clinical Immunology Unit, Department of Clinical and Experimental Medicine, University of Messina, 98125 Messina, Italy; 2 Cardiac/Pulmonary Rehabilitation, ASST Pini/CTO, Milan, Italy; 3 Istituto per le Risorse Biologiche e le Biotecnologie Marine (IRBIM) CNR Messina, Messina, Italy

**Keywords:** *Moraceae*, *Morus*, Mulberry, Cross-reactivity, Respiratory allergy, Food allergy

## Abstract

Mulberry is a plant belonging to the family *Moraceae*, and genus *Morus*. Allergic sensitization to mulberries has been reported as both food allergy or respiratory allergy, and cross-reactivity between mulberries and other pollens or fruits was described. Clinically, in the articles reporting mulberry allergy, the reactions included respiratory allergy, airborne contact urticaria, anaphylaxis, oral allergy syndrome, and food induced urticaria. As far as cross-reactivity is concerned, the allergens identified thus far in mulberries include pathogenesis-related (PR)10 proteins, with sequence identity to Bet v 1 from birch, lipid transfer (LTP)1 proteins with identity with LTPs from Rosaceae family plants, panallergens groups, and also ubiquitin-like protein and cystatin-like protein. The two latter proteins account for cross-reactions with *Parietaria judaica* and *Olea europaea*. Such large cross-reactivity warrants to pay particular attention to the risk of systemic reactions to foods, particularly in subjects sensitized to birch, parietaria or olive pollens. In fact, the increasing use of mulberry as a food product, which is encouraged by its remarkable antioxidant power, expose sensitized patients to possible reactions after ingesting foods, dietary supplements or nutraceuticals containing mulberry. Mulberry allergenicity can vary according to the processing methods used since some allergens are thermostable and other loss their reactivity during heating.

## Background

Mulberry is a plant belonging to the family *Moraceae*, and genus *Morus*. Trees and shrubs of *Moraceae* are primarily tropical, with temperate trees represented by *Morus* (M), *Broussonetia* (B), and *Maclura*. There are 10 *Morus* species, with 3 native to North America, *M. rubra*, known as red mulberry, *M. microphylla*, known as Texas mulberry, and *Maclura pomifera,* also named Osage orange. Paper mulberry, *B. papyrifera* and white mulberry, *M. alba*, are native to Asia, where the latter is grown as food stock for silkworms [1].

Mulberry sensitization has been reported as both food allergy or respiratory allergy to mulberry pollens. There are also cases of cross-reactivity between mulberry and pollens or other fruits. Skin reactivity to red and white mulberries and to paper mulberry is usually strong; all three species have been implicated as local sources of pollinosis.

The aim of this study was to collect and review the published studies and cases of mulberry allergy and the cross-reactivity between mulberry and other allergen sources.

## Methodology

We performed a Medline search, for English-language articles, published until October 2018, using the key words “mulberry” and “allergy”. A second analysis was carried out using the species names of *Moraceae* family.

## Clinical observations

The Medline search identified 10 articles reporting mulberry allergy: 4 studies on respiratory allergy, 1 study on airborne contact urticaria, 2 on anaphylactic reaction, 2 on oral allergy syndrome, 1 on food induced urticaria. Table 1 shows the clinical characteristics of the cases reported.

**Table 1:** Clinical characteristics of mulberry allergy cases

Authors	Sensitivities	Morus type sensitivity	Symptoms after mulberry contact
Zanforlin [5]	House dust mites Cat epithelium	*Broussonetia papyrifera* (China mulberry pollen)	Persistent rhinitis Seasonal asthma
Navarro [7]	Alnus Olea Salsola Chenopodium Plantago Apple Peach Cherry	*Morus alba* pollen *Morus alba* leaf *Morus alba* fruit	Rhinitis Asthma (mulberry leaf) Oral Allergy Syndrome (Rosaceae family) Anaphylactic reaction (mulberry fruit)
Choi [8]	Alder Birch Hazel Oak House dust mites Cat epithelium Dog epithelium	Mulberry fruit (not specified)	Rhinoconjunctivitis Atopic dermatitis Urticaria, chest tightness, breathing difficulty, oral swelling, nasal obstruction (mulberry fruit)
Caiaffa [9] Case 1 Case 2	Grass Olive tree Birch Parietaria Hazel Dermatophagoides farinae Fig Hazelnut Peanut Bean Pea Soybean Maize Apple Peach Cherry Melon Tomato Potato Carrot Celery House dust mites Parietaria Olive tree Peanut Bean Apple Cherry Plum Strawbwrry Orange Fig	*Morus alba* fruit *Morus alba* pollen *Morus alba* fruit	Asthma, rhinitis Lip and oropharyngeal angioedema, pruritus (mulberry) Shortness of breath, lip, tongue and oropharyngeal swelling, pruritus (fig) Perennial rhinitis Urticaria, generalized pruritus, abdominal pain (mulberry, fig)
Wüthrich [10]	Birch Hazel Beech Grass mix Mugworth pollen Apple Peach Celery	*Artocarpus integrifolia* (jackfruit)	Seasonal rhinoconjunctivitis and asthma Oral allergy syndrome
Bolhaar [11] 2 Cases	Birch Grass Apple Hazelnut Peach Peanut	*Artocarpus integrifolia* (jackfruit)	Rhinoconjunctivitis Oral allergy syndrome, dyspnea, hoarseness (case 1) Oral allergy syndrome, abdominal cramps (case 2)
Munoz [12]	Grass House dust mite Olive tree	*Morus alba* pollen	Rhinoconjunctivitis, asthma Erythema and edema of the hands and neck, lips and eyelids angioedema, rhinitis and dyspnea (mulberry)

### Respiratory pollen mediated reactions

The first description dates back to 1971, when Targow [2] reported the impact of mulberry tree on respiratory allergy in South California. In Pakistan, paper mulberry pollen is a major cause of respiratory allergy, and is considered to be a high impact invader in this region [3]. Moreover, mulberry tree pollen is a major aeroallergen in northern regions of India and a predominant source of aeroallergens from January to April in Spain [4]. Exposure to pollen from white mulberry has been reported to cause asthma, allergic rhinitis, allergic conjunctivitis, and symptoms of contact urticaria in Spanish patients. Reports from Italy have also shown a case of pollinosis caused by *Broussonetia papyrifera*, a Chinese mulberry-tree, which is common in Northern Italy [5] and a sensitization to black mulberry fruit in a patient with respiratory allergy [6]. Sensitization to mulberry fruit, pollen and leaves has also been reported in the same patient [7].

### Food induced allergic reactions

Two cases of anaphylactic reaction to mulberry fruit have been reported in patients with a history of respiratory allergy [7, 8].

Oral allergy syndrome have been reported, caused by ingestion of white mulberry [9] and jackfruit [10, 11], in patients with several sensitization to inhalant and other food allergens mostly belonging to Rosaceae family, as well as to fig, a fruit closely related to mulberry.

Moreover, a case of airborne contact urticaria due to white mulberry pollen was also described in a patient with respiratory symptoms. Mulberry airborne contact urticaria was suspected due to the negative patch test to fresh mulberry leaves, the absence of reactions harvesting the leaves outside the pollen season and the in vivo and in vitro sensitization to mulberry [12].

## Molecular components

### Bet v 1 related allergens

In 1997 in a patient with allergy to *Morus alba* pollen, leaf and fruit, two distinct proteins of 10 kDa and 18 kDa were detected in mulberry fruit; the first band was also present in apple and peach extracts and was not identified as profilin; the second was also detected in birch pollen, mulberry leaf and peach extracts [7].

In 2010 a 17 kDa protein has been found in several Moraceae plants, such as fig, mulberry, jackfruit, with a homology of 60%, 56% and 76% respectively, to Bet v1 [13].

Previously, a 18 kDa protein, called WAP18 (winter accumulating 18 kDa protein) was identified in the cortical parenchyma cells of *Morus bombycis* tree; this protein showed high homology to other pathogenesis-related (PR)-10/bet v 1 proteins, such as Mal d 1, Bet v 1, Pru a 1 [14].

In 2014 a similar protein of 17 kDa was detected in a patient with mulberry allergy, also sensitized to birch and with an oral allergy syndrome to peach, plum and apple; the same protein was found in another patient strongly sensitized to birch pollen, with specific IgE to mulberry fruit [8].

Allergy to jackfruit, a tropical fruit belonging to Moraceae family, can be included in birch pollen-related food allergies. In two patients sensitized to this fruit a similar 17 kDa protein with cross reactivity to Bet v 1 was detected [11], although a previous study showed no cross reaction between jackfruit and Bet v 1 and Bet v 2 [10].

### Ns-LTP

Mor n 3, the first allergen identified in the *Morus* genus by Ciardiello et al. is a 9 kDa protein with high sequence identity with other nsLTP [15]. The highest sequence identity, with a rate of 75%, was observed with Fra a 3, the strawberry nsLTP, followed by Pru p 3 (70%), Mal d 3 (70%), Cor a 8 (62%), Art v 3 (61%), Par j 2 (30%). In vivo tests on ns-LTP allergic patients, mostly Pru p 3 positive, showed a high rate of skin reactivity to Mor n 3 (88%), even in subjects who have never ingested mulberry before.

Moreover, Mor n 3 shows a strong cross-reactivity with other nsLTP; indeed, IgE inhibition test showed a complete inhibition to nArt v 3 and Cor a 8 and a very high inhibition to nPru p 3 [15].

### Other allergens

In *Broussonetia papyrifera*, also known as Chinese mulberry or paper mulberry, two pollen allergens, of 72 kDa and 15 kDa were identified [16]. Another study on paper mulberry pollen a 10 kDa protein was reported as the major allergen, cross-reacting to white mulberry pollen; no identity with Bet v 1 or nsLTP allergens was shown [17]. A recent study on *Morus alba* pollen reported a 14–15 kDa double band, identified as profilin and three new allergens: a nsLTP of 10 kDa (nsLTP-10), a 8 kDa ubiquitin like-protein and a 7 kDa cystatin-like protein. Moreover, nsLTP-10 was found to cross-react with Par j 1 and Par j 2 but not with Pru p 3. Immunoblotting-inhibition analysis revealed also a cross-reactivity between the other two new allergens and protein present in *Parietaria judaica* and *Olea europaea* pollens [18].

## Discussion and conclusions

Allergic reactions to mulberry have been frequently reported for many years. The allergic manifestations described above are quite varied and mainly include asthma or rhinitis induced by mulberry pollen while the reported episodes of anaphylaxis, food allergy and oral allergic syndrome are less frequent. Mulberry pollen has to be considered an important respiratory allergen in many areas. This has led to great attention in certain areas such as US, Spain or Asia versus mulberry pollen due to the numerous cases of rhinitis or asthma. Several studies have analyzed the possible cross reactivity between mulberry and other pollens or fruits, with slightly discordant results. In fact, Navarro [7] first hypothesized a possible correlation between mulberry allergen and birch, in particular with Bet v 1. Later, a mulberry allergen of 17 kDa, the same weight of PR10 protein, with sequence identity to Bet v 1.

Another allergen identified, Mor n 3, is a nsLTP1, cross-reacting with other nsLTP.

Other allergens cross-react with *Parietaria judaica* and *Olea europaea*.

The identification of these new mulberry allergens is of considerable importance. In fact, even in areas where mulberry tree is uncommon, there may be cases of mulberry reaction due to cross reactivity with Bet v1*, P. judaica* or *O. europaea*. Although the most reported reactions are caused by mulberry pollen, it cannot be excluded that mulberry ingestion can cause a reaction due to cross-reactivity with other pollen allergens.

Nowadays mulberry is used not only as fresh fruit, but also food products and for therapeutic purpose, due to the antioxidant effects of phenolic compounds, flavonoids and anthocyanins. Therefore, mulberry leaves and fruits are used for they immunomodulatory and antiinflammatory effects, antibacterial activity and hepatoprotective and cardioprotective functions, for the treatment and prevention of several conditions such as diabetes mellitus, obesity, atherosclerosis, inflammation [19, 20].

Mulberry processing for extract production causes the loss of polyphenols, anthocyanins, and antioxidant capacity [21]; therefore, optimization of food processing aimed to preserve the beneficial effects could improve the quality of the final product. In this context, an alternative to conventional thermal processing, as high hydrostatic pressure method has shown to minimize antioxidant loss [22].

Even food allergenicity and immunoreactivity can change during processing, particularly during heating. It is know that thermal processing decreases PR-10 proteins allergenicity; on the contrary nsLTP and seed storage proteins have heat-stable allergens [23]. Therefore, although during thermal processing some mulberry allergens can loss their reactivity, nsLTP allergenicity can remain. Moreover, the search of alternative processing methods to avoid antioxidant loss during heating, can lead to the persistence of immunoreactivity of thermo-labile allergens.

In conclusion, based on the currently common use of mulberry as a food product, possible reactions after ingesting foods, dietary supplements or nutraceuticals containing mulberry, in subjects sensitized to birch, parietaria or olive pollens, must be taken into account. Moreover, better information on the role of processing methods on the allergenicity can help, in the future, to develop mulberry products with low allergenic potency.

## Data Availability

Data and materials presented are available in the manuscript and are stored in the Allergy and Clinical Immunology Division at University Hospital of Messina.
